# Use of Natural Agents and Agrifood Wastes for the Treatment of Skin Photoaging

**DOI:** 10.3390/plants12040840

**Published:** 2023-02-13

**Authors:** Melania Parisi, Mariavittoria Verrillo, Maria Antonietta Luciano, Giuseppina Caiazzo, Maria Quaranta, Francesco Scognamiglio, Vincenzo Di Meo, Alessia Villani, Mariateresa Cantelli, Lucia Gallo, Giovanna G. Altobelli, Serena Poggi, Riccardo Spaccini, Gabriella Fabbrocini

**Affiliations:** 1 Section of Dermatology, Department of Clinical Medicine and Surgery, University of Naples Federico II, Via Pansini 5, 80131 Naples, Italy; 2 Centro Interdipartimentale di Ricerca per la Risonanza Magnetica Nucleare per l’Ambiente, l’Agroalimentare, ed i Nuovi Materiali (CERMANU), Università di Napoli Federico II, Via Università 100, 80055 Portici, Italy; 3 Department of Agricultural Sciences, Università di Napoli Federico II, Via Università 100, 80055 Portici, Italy; 4 Department of Advanced Biomedical Sciences, Università degli Studi di Napoli Federico II, Via Pansini 5, 80131 Naples, Italy

**Keywords:** skin aging, dermatological application, natural bioactive compounds, antioxidant properties, recycled biomasses, humic substances

## Abstract

Photoaging is the premature aging of the skin caused by repeated exposure to ultraviolet (UV) rays. The harmful effects of UV rays—from the sun or from artificial sources—alter normal skin structures and cause visible damage, especially in the most exposed areas. Fighting premature aging is one of the most important challenges of the medical landscape. Additionally, consumers are looking for care products that offer multiple benefits with reduced environmental and economic impact. The growing requests for bioactive compounds from aromatic plants for pharmaceutical and cosmetic applications have to find new sustainable methods to increase the effectiveness of new active formulations derived from eco-compatible technologies. The principle of sustainable practices and the circular economy favor the use of bioactive components derived from recycled biomass. The guidelines of the European Commission support the reuse of various types of organic biomass and organic waste, thus transforming waste management problems into economic opportunities. This review aims to elucidate the main mechanisms of photoaging and how these can be managed using natural renewable sources and specific bioactive derivatives, such as humic extracts from recycled organic biomass, as potential new actors in modern medicine.

## 1. Introduction

The aging of the skin has fascinated researchers for decades, not only to ultimately prevent wrinkle formation but also because the skin represents an excellent and accessible model organ, allowing the study of intrinsic and extrinsic coordinated factors contributing to the complex phenomenon of aging [1]. Skin problems involving dryness, fine wrinkles, and gradual dermal atrophy are related to intrinsic aging, while extrinsic aging is determined by environmental promoters, exemplified by smoking, poor diet, sun exposure, and environmental pollution, which cause coarse wrinkles, a loss of elasticity, laxity, and rough appearance [2,3]. Many relevant histological changes occur in basal cell layer when intrinsic aging is observed. In this context, advancing age induces the reduction of cell proliferation in the basal layer, the thinning of epidermis, the decrease in contact surface between dermis and epidermis, and, consequently, the reduction of the exchange surface for nutrition delivery to the epidermis [4,5]. The decrease in proliferative capacity to skin cells, consisting of keratinocytes, fibroblasts, and melanocytes, is associated with the cellular senescence process [6]. As early as 1969, it was proposed that long-term exposure to solar ultraviolet (UV) radiation is the primary factor of extrinsic skin aging and is referred to as photoaging [7,8]. the photoaging of the skin is a complex biologic process affecting various layers of the skin with the major damage seen in the connective tissue of the dermis. Clinically, photoaging is characterized by wrinkles, laxity, a leathery appearance, increased fragility, blister formation, and impaired wound-healing [9]. The preservation of all skin layers has taken an important role in social welfare, driving the development of new and innovative products by pharmaceutical and cosmetic industries [10]. Allied to skincare is the rising importance placed on natural products, sustainably obtained via methods that have less environmental impact [11]. In fact, the principle of sustainable practices and the circular economy favor the use of bioactive components derived from recycled biomass. The indications of the European Commission support the reuse of various organic biomasses and organic wastes in different productive sectors [12]. This review aims to focus on the clinical and histological features of photoaged skin and then provide useful insights on recent findings regarding the antiaging properties of natural substances.

Scientific advances highlight the potential beneficial dermatological effects of bioactive compounds derived from renewable sources, such as plants and agri-food residues. A particular challenge is represented by the application of humic substances isolated from recycled biomasses, such as compost, with defined structural activity correlations. Within a circular economy strategy, this research topic is a step forward towards the feasible exploitation of cost-effective and environmentally sustainable approaches that act as achievable cutting-edge technologies in the pharmaceutical and cosmetic sectors.

## 2. Photoaging: Features, Causes, and Effects

The skin is a constantly developing organ. At a young age, there is a balance between the synthesis and degradation of collagen. Over time, photoaging is accelerated by drastic changes in the activity of fibroblasts with a consequent failing in the recovery of collagen, thus fostering the aging process [13]. The dermis is the largest part of the skin and supports the cutaneous vascularization for the transport and transfer of oxygen and nutrients. Most of the dermis is composed of a collagenous extracellular matrix, which confers mechanical strength, resilience, and elasticity to the skin. These functions are altered in both chronologically aged and photoaged skin. Alterations to the organization and structure of type I collagen, the most abundant structural proteins present in the skin, is a hallmark of chronologically aged and photoaged human skin [14]. Biochemical studies have shown that reduced collagen synthesis and renewal is related to the severity of photodamage [15] and individual’s age [16,17]. The progressive fragmentation of the dermal collagenous extracellular matrix has important consequences: it decreases the overall strength of the skin, favors wrinkle formation, and creates a microenvironment that facilitates tumor formation and progression [14].

### 2.1. Pathogenesis of Photoaging: The Role of UV Radiation

Photoaging is marked by a development of different skin changes produced by exposure to ultraviolet (UV) light. The excess of UV energy may lead to the premature aging of the skin, superimposed on the modifications caused by physiological chronologic aging [18]. UV radiation (UVR) can penetrate the external skin layer and interact with skin cells, both fibroblasts and keratinocytes [19]. Depending on the wavelength and carried energy, there are three types of UVR, namely as UVA (320–400 nm), UVB (280–320 nm), and UVC (100–280 nm) [20]. Since the UVC type of radiation is absorbed into the stratosphere, the solar radiation on the earth’s surface is made up of a combination of A and B ultraviolet rays (UVAs and UVBs). Although UVAs are more abundant in sunlight, UVBs have higher energy and are responsible for sunburn. Due to its shorter wavelength, most of the UVB energy is absorbed by the epidermis, while a small percentage hits the papillary dermis [21,22]. Conversely, UVA rays can penetrate deeply into the epidermis and their impact is currently acknowledged as a determinant co-activator in skin photoaging and in the development of melanoma skin cancer [23]. The investigation of photo-carcinogenesis in animal tissue has provided evidence that UVAs play a significant role in photodamage, photoaging, and carcinogenesis.

UVA radiation induces a range of physiological photoaging reactions, including chronic inflammatory signaling, immunosuppression, epidermal thickening, dermal matrix degradation, solar elastosis, the reduction of skin barrier power, and the alteration of tissue homeostasis [24]. Generally, the skin-cell damage of UVAs depends on a direct inflammatory reaction and, indirectly, through induced oxidative stress [25]. The DNA damage has been ascertained as a one of the most serious consequences of excessive skin exposure to UV light. The direct destructive mechanism of UVB and UVA action on DNA molecules is related to the amount of energy absorbed by the base pairs in the DNA chain [26]. Research findings on oxidative processes have documented UVA light to be associated with the generation of intracellular reactive oxygen species (ROS), leading to the peroxidation of barrier lipids whose degradation heavily compromise skin barrier function [27]. According to Fisher et al., the primary mechanism by which UV irradiation boosts the molecular responses is based on the release of ROS components [28]. These highly reactive compounds, comprising superoxide anion, peroxide, and singlet oxygen, at increased concentrations, can denature the main proteins that build up the skin, collagen, and elastin [28]. Furthermore, the metabolic surplus of ROS within the tissues damages the DNA and contributes to skin mutagenesis and carcinogenesis [29]. This process involves the peroxidation of polyunsaturated fatty acids (PUFA) in the skin membrane and the subsequent formation of DNA adducts, 8-hydroxy-2′-deoxyguanosine (8-OhdG), identified as a marker of the mutagenic factor for oxidative DNA damage [30,31].

Immunological reactions, inflammatory disorders, skin pigmentation, and wound-healing prompt the production of different metabolites [32,33]. Ultraviolet irradiation also activates the transcriptional sequence from NF-κB to proinflammatory cytokines genes that include interleukin (IL)-1b, TNF-a, IL-6, IL-8, and various adhesion molecules [34]. The inflammatory skin response to UVA radiation triggers the upregulation of cyclooxygenase and lipoxygenase enzymes, thus promoting the release of prostaglandin-F2α (PGF2α) and 12-HETE from arachidonic acid (AA). Several studies pointed out that the induction of matrix metalloproteinases (MMP) has a major influence on the pathogenesis of photoaging. In fact, UV light induces a wide variety of MMPs from an ever-increasing family [35]. The ROS issued by cell response to UV radiation are prime additional mechanisms involving either the increase of cell surface growth factors or the activation of cytokine receptors and/or of nicotinamide adenine dinucleotide phosphate (NADPH) oxidase [36]. Moreover, ROS are also able to stimulate the mitogen-activated protein kinase family (MAPK), precursors of the activator-1 protein (AP-1), which regulate the transcription of different proteins pertaining to the MMP group, identified as MMP-1, MMP-3, and MMP-9, which are employed in progressive collagen degradation [37].

These MMPs can be streamlined by both UVB and UVA light [38]. Each MMP interacts with specific components of the dermal matrix proteins; MMP-1 cleaves collagen types I, II, and III, while MMP-9, also called gelatinase, is involved in the lysis of collagen type IV, V, and gelatin [39]. Therefore, UV-induced MMPs degrade the collagen of the skin and thus compromise the structural integrity of the dermis. The accumulated fragments arising from collagen deterioration are likely important contributors to the phenotype of photoaged human skin [40].

### 2.2. Photoaging and Skin Cancer

The overload of UV radiation determined by chronic sun exposure induces photoaging and epithelial skin tumorigenesis. Photoaging processes increase ROS concentration, inflammation reactions, DNA damage, and, in some cases, the change in skin cell structures and functions [41]. Skin exhibits various antioxidant defense mechanisms that can override the unfavorable or prejudicial step-up of ROS and free radicals. However, higher doses of or exposure frequency to UV radiation may overwhelm cellular resilience capacity, thereby rousing the oxidative stresses and structural cleavages of peptides and lipids components [42,43]. Generally, skin cancers are related to a combination of both genetic and environmental risk variables. Light colored phototypes, light colored eyes, blond or red hair, and family history of skin cancer, as well as the presence of genetic variants or mutations, are usually correlated with a rise in the probabilistic incidence of melanoma [44].

The incidence of skin cancer has increased and worsened worldwide over the last few decades, leading to it being considered “a major public health problem”. Sun-sensitive and faired-skin populations are at a major risk of developing skin malignancies with a higher prevalence among males and patients aged over 50 years. Basal cell carcinoma (BCC), squamous cell carcinoma (SCC), and melanoma (MM) are, in order of incidence, the most common types of skin cancer, with actinic keratoses (Aks) being the precancerous lesions most frequently found in patients with photodamaged skin [45,46]. Of these, melanoma is the most aggressive, responsible of most deaths from skin cancer when not diagnosed at an early stage. Ultraviolet immunosuppression is considered a crossroad event in skin carcinogenesis [47]. UV exposure adversely affects the skin’s immune system through three different processes: (i) diminishing antigen-presenting cell function, (ii) inducing immunosuppressive cytokine production, and (iii) modulating contact and delayed-type hypersensitivity reactions [48]. BCC and SCC development have been mostly associated with chronic cumulative UV exposure, whereas intermittent exposure and a previous history of melanoma are major risks of melanoma [49]. Indeed, patient education in sun protection and correct information could be important sources for skin cancer prevention.

### 2.3. Photoaging: Solar Lentigo and Melasma

Aggregated skin damage via aging and ultraviolet (UV) radiation accelerates acquired skin pigmentation [50]. Melanin pigments are set up in the epidermis by highly specialized cells, the melanocytes, which are neural crest-derived cells that, during embryogenesis, migrate as melanoblasts into the epidermis and hair follicles via the mesenchyme. Brown–black eumelanin and yellow–red pheomelanin are two types of melanin produced by melanocytes. Eumelanin plays a photoprotective role by limiting the penetration of UV rays into the epidermis and intercepting ROS compounds. In contrast, pheomelanin is not photoprotective, being instead highly phototoxic and supporting the unrestrained or uneven release of ROS with obvious harmful consequences [51,52]. Solar lentigo, known as one of the symptoms of photoaging, is a brownish pigmented macula with irregular borders and a size comprising a few millimeters present in light-exposed areas on the hand, face, and upper back. In the epidermis of actinic lentigo, melanin mainly accumulates in the basal layer [53]. Such lentigo is also characterized by deep epidermal invaginations that form club- or bud-shaped extensions in the dermis. Melanocyte activation is controversial, as their density along the dermal epidermal junction has been found to be similar in terms of lesions compared to perilesional skin [54,55]. It is of interest that transcriptomic studies revealed multiple molecular alterations, notably the dysregulation of keratinocyte proliferation and the differentiation and modifications of the dermal extracellular matrix, suggesting that keratinocytes and the dermal compartment could play a crucial role in the physiopathology of lentigines [51]. Some studies supposed that lentigo ageing patterns may correspond to specific epidemiological contexts [56]. It seems to develop preferentially in dark-skinned Caucasians who have repeatedly been exposed to intermittent and intense sun irradiation throughout their lives and have often developed solar lentigo on their upper backs at an relatively early age, whereas the “prominent wrinkling” pattern, characterized by wrinkles, laxity, and atrophy, is known to affect light-skinned people and smokers who have had a continuous excess of exposure throughout their lives. Solar lentigo, common in ageing patients, is a macular hyperpigmented skin lesion, usually flat with a well-defined border, that results from chronic exposure to ultraviolet irradiation. Although solar lentigines are not premalignant lesions, they may be bothersome to patients and are commonly considered to be a cosmetic problem [57].

Another hallmark sign of photoaging is represented by melasma. High incidence is found in populations living in intertropical areas or in elevated altitudes, where there is greater exposure to UVR [58]. The involvement of UVB and UVA is demonstrated by the reduction of both the intensity and the incidence of the diseases due to the use of broad-spectrum sunscreen [59]. Melasma is a disorder of skin pigmentation characterized by the development of asymmetrical, hyperpigmented macules in sun-exposed areas, especially the upper lip, the cheeks, the forehead, and neck. It especially affects women between 20–45 years old and with IV through VI Fitzpatrik skin type [60]. The pathogenesis of this disorder may be attributed to massive levels and/or incorrect forms of sun exposure, genetic predisposition, pregnancy, oral contraceptives, and drugs. Centro-facial, malar, and mandibular are the three main clinical patterns of melasma, while epidermal, dermal, mixed, and intermediate are the four types of melasma observed in Wood’s light (320–400 nm) examination [61]. Histological analysis has shown increased solar elastosis, basement membrane disruption, rete ridge flattening, increased microvasculature, the infiltration of mast cells, and subclinical inflammation [62]. Biochemical pathways of melanogenesis process are based on the hydroxylation of tyrosine and the formation of L-dihydroxyphenylalanine (L-DOPA) which stimulates the production of eumelanin and hydrogen peroxide (H_2_O_2_) as a byproduct [63]. The generation of H_2_O_2_ in the melanocyte produces oxidative stress, leading to cell destruction. However, in normal conditions, the melanocyte has certain antioxidant molecules, such as catalase and glutathione, which can reduce H_2_O_2_, causing a condition of equilibrium in all redox reactions in the melanogenesis pathway [64]. Tyrosinase plays a central role in preventing adverse skin conditions and hyperpigmentation. Some polyphenols extracted from recycling vegetable biomasses exhibited an inhibitory effect against tyrosinase. For example, quercetin halted the activity of the enzyme tyrosinase through the chelation of copper atoms included as a cofactor in its enzymatic structure which degrade the elastin in the extracellular matrix, thus fostering the photoaging process [65]. A combination of polyphenols and flavonoids in the same bioactive extracts induce a greater anti-melanogenic effect, reducing hyperpigmentation and preventing melanogenesis.

Many genes are known to be dysregulated by the Wnt pathway produced by fibroblasts [66]. These results suggest that melasma affects not only melanocytes but also the dermal compartment and that long-term exposure to solar radiation may be a key process in its development. For this reason, patients with fair skin phototypes tend to develop melasma at a younger age [67]. In contrast to darker phenotypes, melanin is employed as a natural photoprotectant by delaying the onset of melasma. Reviewing epidemiological and pathophysiological data, Passeron and Picardo proposed the new paradigm of melasma as being a photoaging skin disorder affecting genetically predisposed individuals [68].

## 3. Photoprotective Natural Compounds: Chemical Features and Medical Application

**Figure 1:**
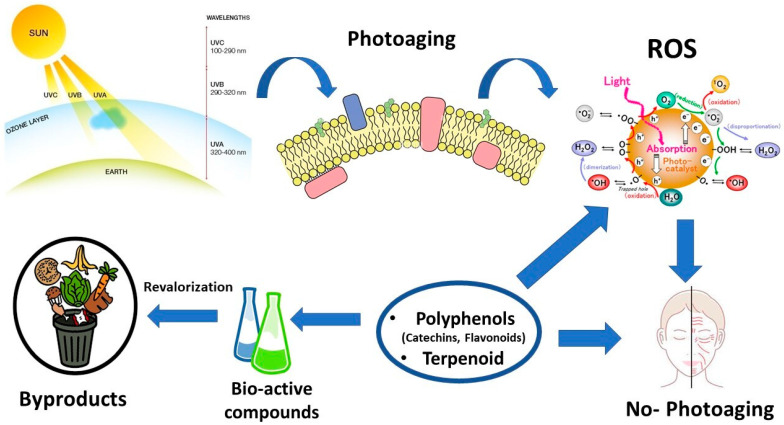
Effect of different bioactive compounds to suppress the photoaging process.

Photoprotection is a process that minimizes the damage to different organisms, either human or plant, when exposed to UV radiation [69]. Several photoprotective compounds isolated from plants or fungi, ranging from scytonemins, mycosporines, mycosporine-like amino acids, phenyl propanoids, flavonoids, and melanins, have been identified as effective contrasting agents of photoaging damage [70]. Some of the potential mechanisms of photoaging inhibition processes triggered by natural bioactive compounds are summarized in Figure 1.

Generally, secondary metabolites were recognized as responsible for plant adaptation and survival, especially under non-favorable conditions [71]. Their functions include, for example, UVB screening compounds, growth hormones, and signaling compounds. The production of natural secondary metabolites in higher concentrations is an adaptive mechanism in response to enhanced UVB exposure [72]. Secondary metabolites are synthesized in plants as stress-response mechanisms that are accumulated in epidermal plant layers and act as sunscreens to protect the underlying sensitive tissues from the damaging effects of UVB. However, prolonged exposure to UVB might lower their protective potential, probably by reducing overall photosynthate production [73]. Phenylpropanoids and their glycosides are synthesized in plant cells through the phenylpropanoid pathway immediately upon exposure to UV. They exhibit sunscreen properties due to their molecular structure characterized by the presence of condensed aromatic 5- and 6-carbon rings with multiple OH groups that effectively absorb UVA + UVB [74]. Moreover, since the presence of glycosyl moieties in secondary metabolites provides increased photostability, many of the glycosylated metabolites are not susceptible to UVA destruction. Different natural compounds pertaining to catechins, flavonoids, and terpenoids employed in plant protection reduce the effects of reactive oxygen species (superoxide anion-radicals, hydroxyl radicals, hydrogen peroxides, and singlet oxygen) produced by UV reaction with organic compounds in the presence of molecular oxygen [75]. Conversely, terpenoids use UV energy to promote biological reactions based on free radical-driven photochemical reactions [76]. In recent years, beauty care products have focused on compounds with potential antioxidant activity of natural origin that are able to induce MMP and tyrosinase inhibitory activity in order to reduce ROS caused by UV radiation and to delay skin aging [77]. Tyrosinase is the enzyme that catalyzes the oxidation of tyrosine to L-Dopa and dopaquinone, a key intermediate in the synthesis of melanin, the pigment responsible for skin color. The phenomenon of hyperpigmentation is induced by the anomalous accumulation of melanin pigments in the skin. Overexposure to UV radiation produces the unnatural synthesis of melanin, resulting in skin pigmentation. Tyrosinase inhibitors may function as candidates to control hyperpigmentation or skin bleaching due to the role of tyrosinase in melanogenesis processes [67,78]. Several signaling pathways are associated with melanin synthesis. In particularly, the cAMP pathway is a major regulatory linkage mechanism that enhances the expression of the microphthalmia-associated transcription factor MITF, which regulates the expression of tyrosinase required for melanogenesis [68,79]. Previous studies have observed that certain algae fractions affect melanin synthesis and cellular tyrosinase activity by upregulating the CREB, PKA, and cAMP pathways. Furthermore, polysaccharides from plant sources may play an important role in inhibiting the activity of collagenase and elastase enzymes involved in photoaging processes. The inhibition of sulfated polysaccharides through vegetable waste against intracellular collagenase and elastase, regulating NF-κB pathways, AP-1, and the MAPK of HDF cells treated by UVB irradiation was previously discussed [69,80]. Conversely, fucoidans extracted by recycling biomasses can inhibit the action of elastase and collagenase by reducing the expression of MMP 1 in skin fibroblast cells and at the same time increasing the expression of procollagen type 1 and the attenuated UVB-induced production of inflammatory cytokines, collagen breakdown, and mast cell infiltration [70,81]. Photoprotective pigments are mostly located in peripheral tissues or accumulated in dermal and/or vascular tissues in order to increase the efficiency of UV absorption. The efficient extraction and careful purification of photoprotective compounds are basic protocol requirements to study the characteristics of UV-absorbing natural resources [82]. Pressurized liquid extraction (PLE), solid–liquid extraction, and Soxhlet extraction are common and viable analytical techniques used to isolate unaltered UV-absorbing species from the biological samples. Additionally, other methods, such as microwave-assisted extraction (MAE), ultrasound-assisted extraction, and supercritical fluid extraction are becoming increasingly popular [83]. Conversely, enzymatic hydrolysis or acid hydrolysis is used in conjunction with a lignin biopolymer that is considered a valuable natural source of bioactive products in photoaging processes. Natural lignin is a common structural component of herbaceous and woody higher plants, accounting for 15–40% of its dry weight [84]. Commercially available lignin, called technical or industrial lignin, is the by-product of the pulping processes. The task of lignin in plants is to strengthen the cell wall and protect cellulose fibers from UV radiation throughout plant life. In addition to UV protection functions, lignin is an excellent antioxidant due to its free radical scavenging capacity, providing effective thermal and oxidation stability when applied in cosmetic blending without observable cytotoxicity effects [85]. The investigation of lignin nanoparticles or composite films as UV light filters and antibacterial agents confirmed the function of this natural substance in photoaging processes [86]. Plants’ UV protection systems may be compared to the human cutaneous photochemical defense based on pheomelanin and melatonin. Proteins rich in aromatic amino acids exhibited photoscreening activities, while eumelanin, porphyrins, flavins, and hemoglobin behaved as photosensitizers molecules [87]. The efficacy of the human barrier against UV rays can be related to photoprotector/photosensitizer balance, which is susceptible to gradual deterioration through the aging progress [88]. In dermatological fields, sunscreen lotions and creams are the most popular skincare products that are recommended as primary preventive measures for UV-induced skin disorders [89]. Organic sunscreens contain compounds with extended chromophores comprising mononuclear or fused aromatic rings along with olefins and carbonyl functionalities. Extensive research is being carried out to incorporate various naturally occurring organic materials, such as UV filters, since they are renewable, non-toxic, biocompatible, and efficiently absorb UV radiation [90,91].

## 4. Agro-Food Residues

Agro-food residues have long been considered as unavoidable leftovers of the agro-industrial sector to be managed from an environmental point of view as depreciated organic waste. However, following the quick diffusion of circular economy concept in different productive sectors, these residual biomasses and by-products are currently regarded as a viable and promising source of valuable natural organic molecules to support the development of breaking innovative products in the field of biopesticides and biofertilizers, as well as for nutraceutical and pharmaceutical research and innovation strategies. For instance, a large number of projects have been funded in the last three years by the Bio-Based-Industry JU European program (https://www.cbe.europa.eu, accessed on 13 December 2022) to foster the sustainable and cost-effective production processes of natural and healthy ingredients for nutraceutical and cosmetic applications [92].

Despite the interest in bio-based molecules for cosmetic and pharmaceutical use, the number of products on the market produced through the valorization of food by-products is still somewhat limited and underexploited. It is estimated that the global regenerative medicine market is expected to increase from USD 8.5 billion in 2020 to USD 17.9 billion by 2025 at a CAGR (compound annual growth rate) of 15.9%. The functional cosmetics market, which encompasses antiaging skin products, is expected to grow from USD 3.2 billion in 2021 to USD 4.1 billion by 2026 at a CAGR of 5.2% [93]. A depiction of current share of the global market and estimated future trends is shown in Figure S1 in the Supplementary Materials. Different agro-food wastes and process byproduct residues have been tested for antioxidant and photoprotective activity against UVB radiation. Residual blanch water (BW) from the almond processing industry, enriched at the polyphenolic level, was used to evaluate the antioxidant and the radical scavenging activities and the in vivo photoprotective effect on skin erythema induced by acute UVB exposure in twelve volunteers [94]. Results confirmed the larger presence of antioxidant compounds in industrial BW extracts, mainly represented by naringenin-7-O-glucoside and kaempferol-7-O-rutinoside. The good antiradical activity of the BW extract was positively correlated with the percentage inhibition of the erythema obtained from the formulation of BW, demonstrating an effect against photo-oxidative damage in vivo. The conventional Soxhlet method was used to test the extraction efficacy of five different solvents on the peel residues of two *Citrus* spp. [95]. The outcomes highlighted high absorption at both UVA and UVB regions from all tested extracts, with no relevant negative impact on the antioxidant components, indicating the potential of these fruits as sources of UV protector molecules against harmful UV radiation. Smart technology was designed to isolate the ferulic acid (FA), a powerful antioxidant compound, from brewers’ spent grain in the beer industry and produce an encapsulated nanocomposite for skin application [96]. The outputs demonstrated an effective extraction of ferulic acid and the suitable development of a formulation of ultra-deformable liposomes containing FA, creating a high-value commercial product for the pharmaceutical and cosmeceuticals industry. The obtained nano-formulation enabled the transportation of FA to the deep layers of the skin, while no toxicity on cell lines derived from skin was detected at the tested doses.

Research into biomolecules for skincare and photoaging treatment has highlighted the significant potential of Chitin and Lignin, natural structural biopolymers, which may find extensive application in cosmetic dermatology in order to produce biocompatible tissues at low cost [97]. In fact, these natural biocomposites may be extracted from food waste obtained from the industrial processes of crustaceans and sugarcane, respectively. It has been shown that nano-chitin–hyaluronan (CN-HA) and nano-chitin–lignin (CN-lignin) copolymeric composites may act as either active carriers or ingredients for the treatment of prematurely aged skin. They may bind to its structure, load, and release different active ingredients with antioxidant and anti-inflammatory activities. Alternatively, they may undergo enzymatic metabolization and release glucosamine, acetyl glucosamine, glucose, hyaluronic acid (CN-HA), or polyphenols (CN-lignin), taken from skin cells as raw material and energy that are useful for counteracting aging processes and increasing photoprotective activity [98]. The beneficial use of seafood residues as source of photoprotective ingredients has been proven in a recent study based on the enzymatic degradation of tissues [99]. The amino acids released by hydrolysis, including glycine, leucine, proline, serine, tyrosine, and threonine, were able to reduce the photoaging effects by modulating the deposition of collagen fibers and strengthening the rebuilding of the extracellular component matrix in rats. The authors uncovered that low molecular weight hydrolysates improved the barrier functions of photoaged skin and stimulated the deposition of type I, hydroxyproline, and hyaluronic acid in the dermal layer. Simultaneously, it was observed the inhibition of matrix metalloproteinase-1 in photoaging skin and the stimulated expression levels of elastin and fibrillin-1 had a significant dose response effect.

## 5. Humic Substances

Among natural organic materials acting as bioactive compounds in dermatological application, an innovative outlook is given to the so-called humic substances (HS). HSs are ubiquitous natural organic materials present in soils, sediments, and aquatic environments. They originate from the chemical and biological transformations of organic residues of plant and animal origin combined with microbial by-products [100]. The HSs are some of the largest pools of organic carbon in the biosphere and has a crucial flywheel effect on the geochemical cycles of C, N, and P and acts as a biostimulant and bioactive agent for both plant development and medical treatment [101,102]. Notwithstanding the different origins and developing conditions, humic substances retain basic common molecular features determined by the selective preservation of specific components that promote the formation of the micelle-like assembly of polar and apolar components with a wide molecular weight distribution that display similar conformational properties. These characteristics seem closely linked to the bioactive functionalities expressed by HS [103,104]. Although a large number of studies and applications are carried out under the bioactive influences of plants and micro-organisms, HSs have been also analyzed for their potential use in medical treatments. The majority of research activities deal with either complex organic materials found in mud or aquatic sediments [105,106] or soluble humic fraction represented by the so-called fulvic acid from non-renewable geochemical organic sources, such as peat, oxidized coal, lignite etc., [107,108]. A more limited range of studies has investigated the use of humic substances for cosmetic or dermatological applications [106]. In an in vitro experiment, the ultraviolet-induced skin aging was effectively treated with the use of fulvic acid, which promoted an increase in fibroblast viability and the prevention of collagen degradation [109]. To develop biomaterial for scarless tissue regeneration by suppressing collagen type I overexpression and the stimulation of collagen type III expression, humic acid and alginate were cross-linked to obtain a hydrogel membrane, which was tested as anti-inflammatory and wound-dressing treatment [110].

**Figure 2:**
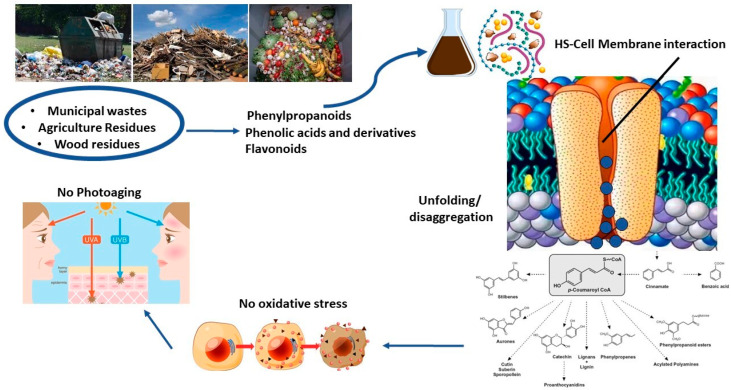
Hypothesized mechanism associated with the potential photoprotective activity of agro-food residues and humic substances.

An innovative objective in the research of natural products for skin treatments is represented by the utilization of humic substances obtained from recycled biomasses symbolized by compost, vermicompost, digestates, etc. Differently to materials of non-renewable origin and the narrow structural variety of geochemical materials, the strategy based on the reuse of processed organic residues may rely on potential unlimited sources, corresponding to a wide range of molecular features and structural compositions. The main research areas that are currently under study concerning compost extracts focus on either the biostimulant activity for aromatic plants to improve the yield and effectiveness of antioxidant and anti-inflammatory metabolites [111,112] or the tailored selection for direct skin application [108,113]. In a recent study, the humic molecules from two green compost types were applied in order to test anti-inflammatory activity and potential dermatological applications as compared to conventional humic material from geochemical origin [113]. The authors found that the humic materials applied to human keratinocyte revealed a general increase in viability, while the gene expression of IL-6 and IL-1β cytokines indicated a significant decrease after humic application, as related to structural properties, suggesting a protective effect of humic matter due to the release of phenolic and lignin components. Previous mechanistic investigations into the protective or antioxidant activity of fulvic acids have uncovered the role of small active fractions made up of bio-organic acids, carbohydrates, and phenol derivatives [98,102,114]. Although structural activity relationships have not yet been elucidated, the current analyses concerning the bioactive functions of humic materials highlight the simultaneous occurrences of synergistic effects related to conformational properties and the inclusion of bioactive molecules (Figure 2). The structural arrangement based on the multilayer stacking of hydrophilic/hydrophobic components held together by non-covalent bonds [115] behave as protective carrier systems that may foster interaction on membrane cells. Upon adsorption, the metastable humic aggregates may undergo a structural rearrangement, thus allowing a release in cellular environment of small bioavailable active molecules [111,112,113].

## 6. Conclusions

This review aims at describing the acknowledged key elements involved in skin photoaging and how these can be addressed and mitigated using natural organic compounds from recycled substances and renewable sources as potential new actors in modern medicine. The mechanisms of photoaging are related to the production of ROS and the appearance of UV-induced DNA damage, as well as to alterations in signal transduction pathways mediated by inflammatory/oxidative stress, inflammatory and immunosuppressive mechanisms, and changes in angiogenesis. Photoprotective strategies include DNA repair through DNA repair enzymes, the removal of ROS with antioxidant agents, and anti-inflammation/immunomodulation with anti-inflammatory agents. Several studies have demonstrated that antioxidants deriving from natural products are particularly effective in the protection of skin against photoaging. The dedicated literature confirms that there are certain natural substances, such as phenylpropanoids or flavonoids, involved in the prevention of photoaging damage. Moreover, the review of the literature suggests that humic extracts applied to human keratinocyte induced a general increase in IL-6 and IL-1β cytokines, suggesting the protective effect of these natural organic materials. Recent scientific advances highlight the potential beneficial dermatological effects of humic matter derived from renewable sources, such as recycled biomasses with defined structural activity correlations. The comprehensive understanding of the specific relationship between the molecular features of natural extracts or derivates and their bioactivity represents an important step forward in supporting the potential exploitation of natural sustainable materials in the medical field.

## Data Availability

The data summary is contained within the article.

## Supplementary Materials

The following supporting information can be downloaded at: https://www.mdpi.com/article/10.3390/plants12040840/s1. Figure S1: Exemplificative data on market trends of antiaging products from web sources.
