# Supplementary material for: Use of Natural Agents and Agrifood Wastes for the Treatment of Skin Photoaging
Source: Plants (Basel). 2023 Feb 13;12(4):840. doi: 10.3390/plants12040840 (PMC9966275; doi:10.3390/plants12040840)
Supplement: Supplementary file 1 [file plants-12-00840-s001.zip › plants-2129456-supplementary.pdf]

Supporting information

Use of natural agent and agrifood wastes to treatment of skin photoaging

by Melania Parisi1, et al.,

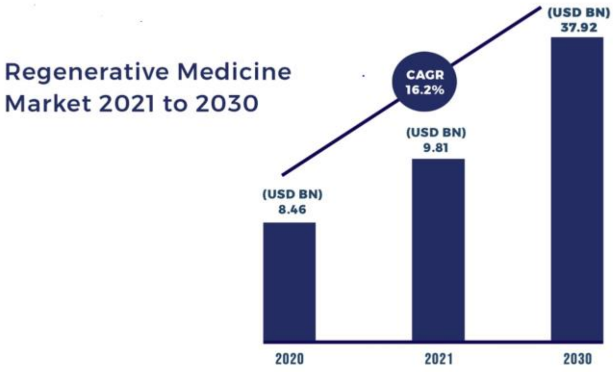

Source: www.precedenceresearch.com

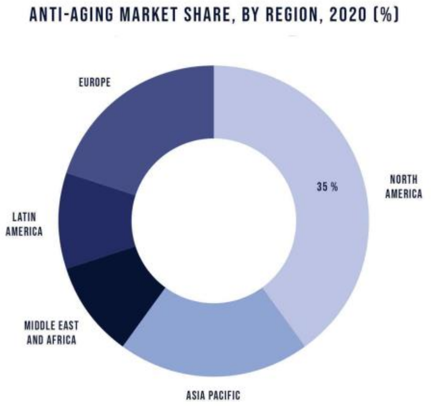

Source: www.precedenceresearch.com

Europe anti-aging market 2018-2027

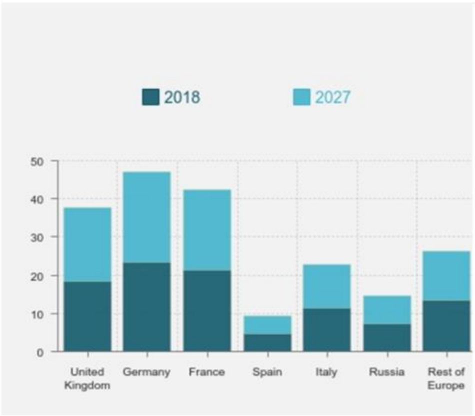

Source: tritonmarkerresearch.com

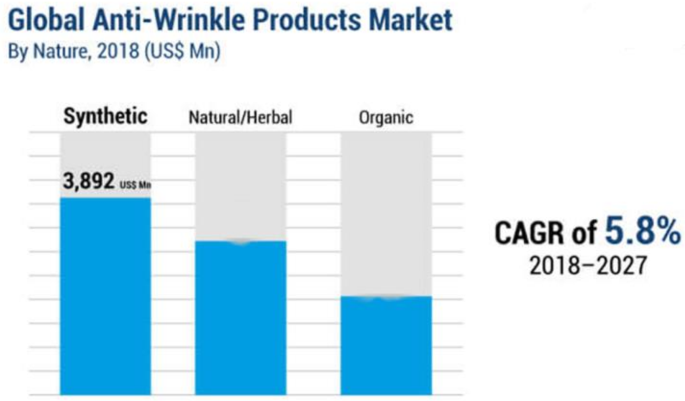

Source: FMI futuremarkerinsights.com

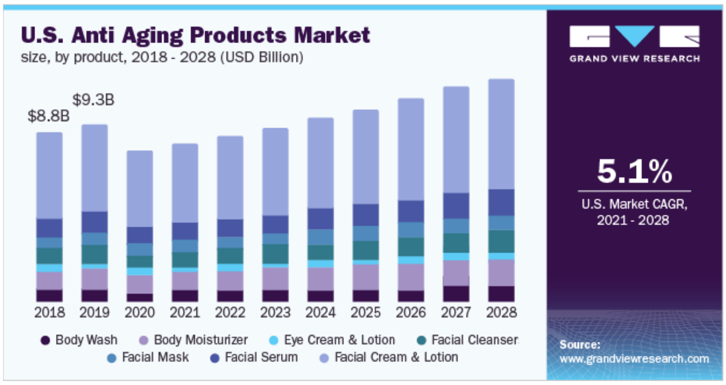

Source:Anti Aging Products Market Size Report, 2021-2028 grandviewresearch.com)

Figure S1: Exemplificative data on market trends of antiaging products from web sources
